# Chromatin dynamics: Nucleosome occupancy and sensitivity as determinants of gene expression and cell fate

**DOI:** 10.46439/cancerbiology.2.024

**Published:** 2021

**Authors:** Jane Benoit, Mahdi Khadem Sheikhbahaei, Jonathan Dennis

**Affiliations:** Department of Biological Science, Florida State University, Tallahassee, FL 32306, USA

**Keywords:** Chromatin remodeling, Nucleosome, Gene regulation, Epigenetics, Adenocarcinoma

## Abstract

The nucleosome, consisting of ~150bp of DNA wrapped around a core histone octamer, is a regulator of nuclear events that contributes to gene expression and cell fate. Nucleosome organization at promoters and their associated remodeling events are important regulators of access to the genome. Occupancy alone, however, is not the only nucleosomal characteristic that plays a role in genome regulation. Nucleosomes at the transcription start sites (TSSs) of genes show differential sensitivity to micrococcal nuclease (MNase) and this differential sensitivity is linked to transcription and regulatory factor binding events. Recently, lymphoblastoid cells treated with heat-killed *Salmonella typhimurium* were shown to exhibit increased MNase sensitivity specifically at genes implicated in immune responses. Increased sensitivity at the −1-nucleosome permitted transcription factor and RNA Pol II binding events. This system illustrates how cytoplasmic signals induce altered chromatin states to produce a specific cellular response to a stimulus. Innate immune activation is a longstanding model for inducible promoters, transcriptional activation, and differential nucleosomal sensitivity in response to immune activation and offers a model that may be largely applicable to other specific cellular responses including viral infection and cancer. Previous work has shown that early transformation events are associated with prolonged nucleosome occupancy changes that are not observed later in cancer progression. Herein, we propose a model in which we suggest that detailed studies of nucleosomal occupancy and sensitivity in response to specific stimuli will provide insight into the regulation of nuclear events in cancer and other biological processes.

## Nucleosome Position is a Classic Indicator of Gene Regulatory Potential

The nucleosome has long been considered a regulator of nuclear events. Consisting of ~150bp of DNA wrapped 1.65 times around a histone octamer, the nucleosome serves as the functional subunit of chromatin [1]. Nucleosomal regulation of eukaryotic transcription was first recognized in the yeast PHO5 gene which is inactivated by nucleosome occupancy at upstream activating sequences. Low phosphate conditions disrupt these nucleosomes leading to gene activation [2–4]. Similar nucleosome-mediated gene regulation is present in the glucocorticoid receptor-activated promoter of the mouse mammary tumor virus long terminal repeat. Glucocorticoid binding results in nucleosome delocalization which modifies a transcription factor binding site to alter gene transcription [5,6]. More high throughput technologies, including DNA microarrays, established a pattern of nucleosome occupancy around transcription start sites (TSSs) characterized by a nucleosome-free region flanked by statistically positioned nucleosomes [7–9]. These studies indicate that nucleosomal promoter architecture and associated remodeling events are key to regulating access to genomic DNA for regulatory factors. These seminal papers provided the foundation for studies of nucleosome remodeling dynamics.

The availability of massively parallel sequencing technologies in the early 2000s allowed for more detailed studies that mapped nucleosome occupancy in a highly time-resolved manner and provided insights into the dynamics of nucleosome remodeling. Time-resolved studies demonstrated widespread transient changes in nucleosome occupancy in response to reactivation of Kaposi’s sarcoma associated herpesvirus (KSHV) to produce a transient intermediate chromatin conformation favorable to an immune response followed by a return to the basal state [10]. Similarly, widespread nucleosome remodeling was shown to be an early event in lung and colon adenocarcinoma progression [11,12] with nucleosomes returning to their basal positions later in the progression of these cancers. Transient nucleosome remodeling appears to be a general feature of genome regulation as similar changes in nucleosome occupancy have also been noted, for example, in VDJ recombination [13], nicotine and cocaine stimulation [14], and the innate immune response in macrophages [15]. In each of these recent studies, the temporary nature of the nucleosome remodeling event suggests a window of opportunity that allows for specific cellular responses to stimuli and potentiate differential genomic regulation.

This type of transient remodeling may also play a role in response to other stimuli including viral infections and resulting immune-mediated pathologies like those seen in acute SARS-CoV-2 infections and persistent systemic symptoms classified as Post-Acute Sequelae of COVID-19 (PACS). Chromatin remodeling complexes are essential for viral infection and replication indicating a direct role for chromatin dynamics during viral infection [16]. Initial remodeling events, triggered by a response to immunomodulatory viral proteins [17], may poise host chromatin and initiate the immune pathophysiology associated with the disease [18]. Changes in nuclear architecture alter immediate gene expression as well as long-term outcomes which may contribute to persistent disease months after viral clearance. Despite a growing understanding of the implications of nuclear architectural changes, the role of these modifications in COVID-19 and viral infections is largely unknown.

## Changes in Nucleosome Sensitivity Dynamics are Linked to Changes in Cell Fate

**Figure 1:**
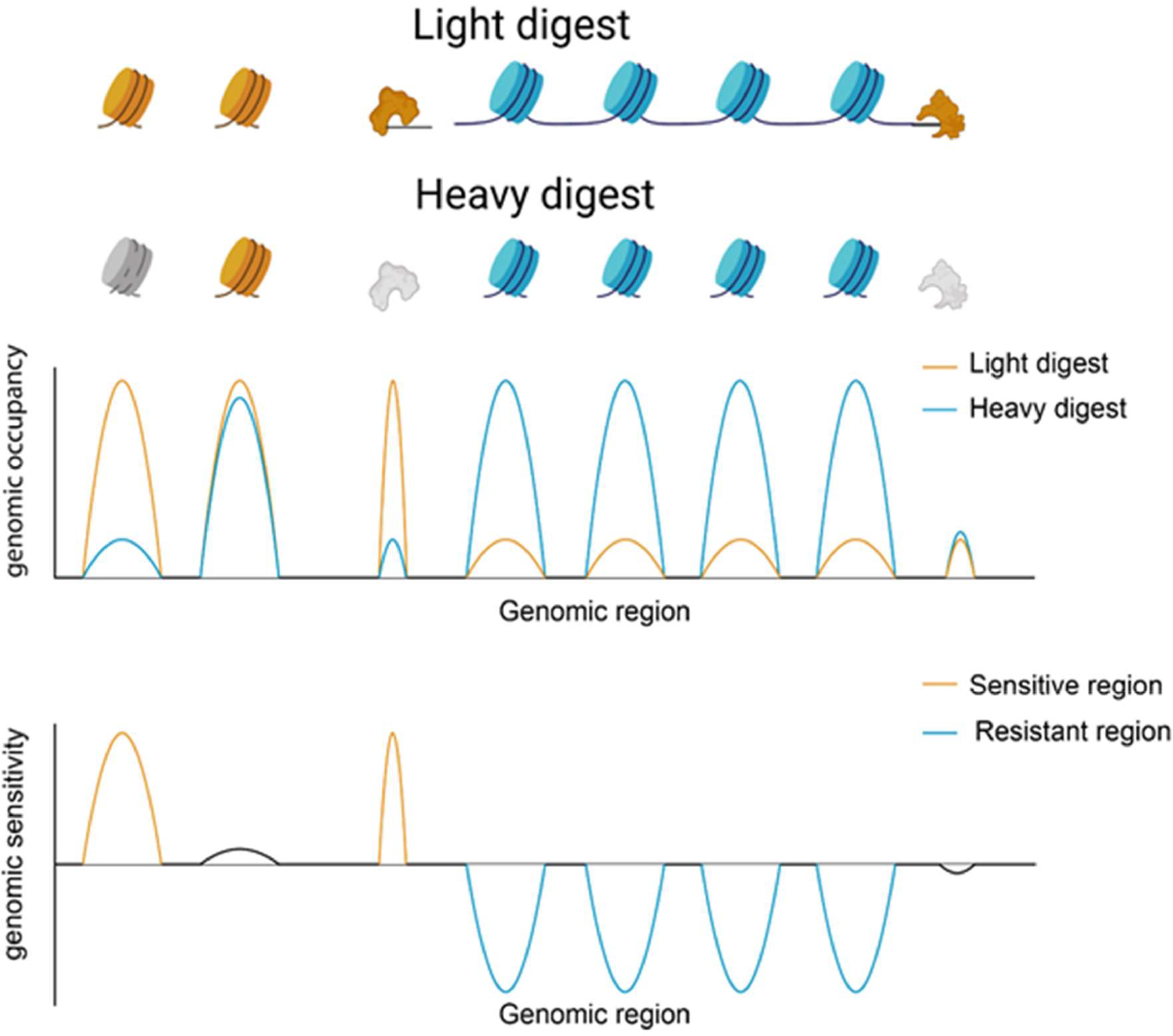
Nucleosome sensitivity to MNase digestion. Sensitive nucleosomes and bound factors, indicated by the color yellow, are preferentially released under light MNase digestion conditions. Resistant nucleosomes, shown in blue, are resistant to light digest conditions and are cut under heavy MNase digestion. Heavy MNase digestion over digests sensitive nucleosomes and bound factors, shown in grey. The middle panel depicts the average normalized number of mapped reads to genomic regions from both heavy and light MNase digestion. In the bottom panel, MNase sensitivity is determined by calculating the log ratio of mapped reads from light and heavy digest conditions.

Nucleosome mapping is often achieved by digesting crosslinked chromatin in micrococcal nuclease (MNase) to produce ~150bp fragments which correspond to nucleosome-protected immune-specific regions [19] (Figure 1). Biochemical properties of nucleosomes differ throughout the genome which alters nucleosome stability and thus sensitivity to MNase digestion [20–22]. Nucleosomes show differential sensitivity to digestion with MNase with a subset of nucleosomes released preferentially at low MNase concentrations while others are released only at higher concentrations (Figure 1). The heterogeneous response to MNase titrations suggests an underlying difference in nucleosome accessibility seen in enzymatic digestion [23]. Although the relationship between MNase sensitivity and physiological DNA accessibility is unclear, differential sensitivity has been linked to altered transcription and regulatory factor binding states [24,25]. Nucleosome occupancy remains unchanged from the canonical promoter structure in GM12878 lymphoblastoid cells stimulated with heat-killed *Salmonella typhimurium.* However, increased sensitivity at the −1 nucleosome in highly expressed genes is observed 20 minutes after stimulation which is consistent with the timeframe for the innate immune response to bacterial stimulation [24,26]. MNase sensitive nucleosomes in stimulated cells flank specific Pol II and immune transcription factor binding sites indicating that early changes in chromatin structure allow for specific genomic responses mediated by regulatory factor and transcriptional machinery binding events [24]. The positioning of sensitive nucleosomes near transcription factor binding sites allows for rapid responses to stimuli to produce highly specific genomic responses associated with cell identity and fate. We suggest that nucleosome sensitivity dynamics are likely a more generalizable characteristic of cell fate and identity than nucleosome positioning alone.

## Nucleosome Dynamics in Cancer Progression

Genomic instability and altered chromatin organization are the hallmark of the transformed phenotype and are associated with cancer progression. Features of chromatin organization (e.g., nucleosome assembly, nucleosome ejection and translocation, and substitution of nucleosome core subunits) are regulated by ATP-dependent chromatin remodeling complexes [27]. Mutations in these complexes are drivers in cancer development [28] and their mutation is frequently observed in many cancer types [29–32]. Aberrant regulatory interactions between these chromatin remodeling complexes and nucleosomes result in altered nucleosome dynamics likely leading to impaired gene regulation and cancer development. This proposition is supported by observations of nucleosomal dynamics in adenocarcinoma.

Early grade adenocarcinomas show widespread nucleosome depletion at transcription start sites particularly in genes associated with chromatin structure and cancer progression. While it is unknown exactly how long this remodeled state persists, an appealing explanation is that this remodeled state persists longer than physiologically expected due to dysregulation of ATP-dependent chromatin remodeling complexes. Consistent with this explanation, differential occupancy is reduced in higher grade tumors [11], hinting that, after some time, the remodeled nucleosome occupancy that is characteristic of early cancer progression returns to basal nucleosome occupancy later in cancer progression through some redundant or compensatory nucleosome remodeling mechanism. High-grade lung adenocarcinoma tissue shows genome-wide changes in nucleosome sensitivity to MNase compared to normal tissues suggesting that canonically positioned nucleosomes in high-grade cancer have differential accessibility [12]. The period of differential accessibility, as indicated by MNase sensitivity, opens a window of opportunity for regulatory factor binding and subsequent alterations in gene expression. Depletion upstream of the −1 nucleosome was noted in genes implicated in nucleosome assembly, metabolic processes, and cancer progression which supported earlier results from a subset of cancer and immune specific genes [11,12]. Changes in nucleosome architecture in early lung and colon adenocarcinomas altered transcription factor binding site accessibility suggesting that differential occupancy may play a role in cancer development [11]. While nucleosome remodeled states observed in early cancer samples eventually return to basal nucleosomal occupancy, changes in nucleosome sensitivity remain after nucleosomes return to basal positions. These observations comport with the relationships observed between nucleosome remodeling and sensitivity in the *Salmonella* example above. The longer-lived nucleosome sensitivity changes explain differential gene activation through altered regulatory factor accessibility in conditions, such as high-grade cancers, where nucleosome occupancy appears largely static.

## Nucleosome Occupancy and Sensitivity as Determinants of Gene Expression and Cell Fate in Cancer

**Figure 2:**
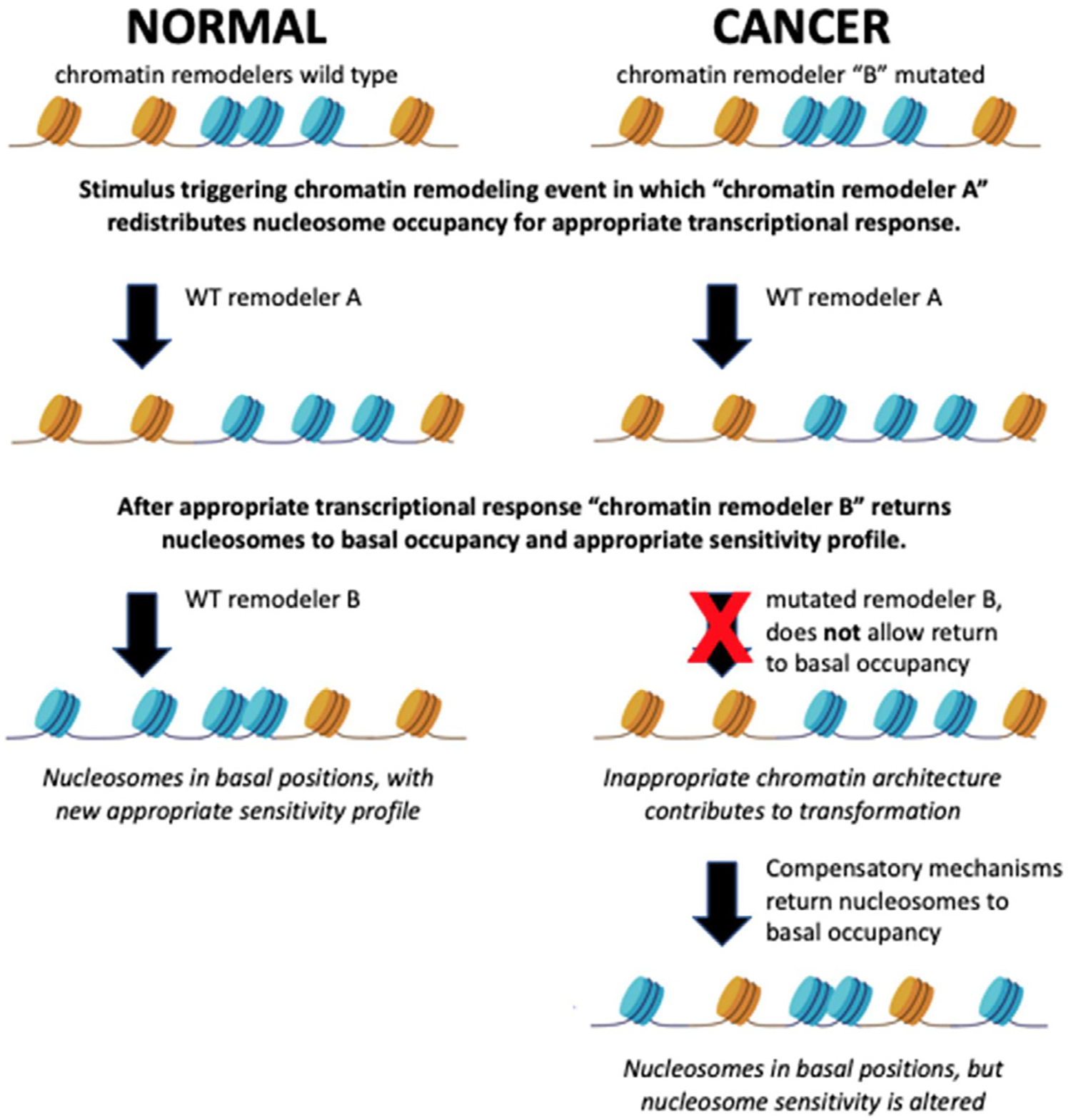
Proposed model for the roles of nucleosome remodeling and sensitivity in oncogenic transformation. Sensitive nucleosomes are depicted in yellow while resistant nucleosomes are shown in blue. Under normal conditions, chromatin remodelers return nucleosomes to basal positions with an appropriate sensitivity profile following a transcriptional response. Cancer cells have mutated remodeling profiles that result in inappropriate sensitivity profiles following transcriptional response.

The above observations suggest that measurements of nucleosome occupancy and sensitivity will provide valuable insights into gene expression and cell fate outcomes in cancer progression. We propose a model to explain these relationships (Figure 2). In this model, the prospective cancer cell undergoes a mutation in a critical chromatin remodeling complex responsible for returning a remodeled nucleosome architecture to basal nucleosome occupancy. Upon receiving a stimulus for some gene regulatory event, the genome is appropriately remodeled for the cell-type specific response; however, the chromatin is not able to return to basal nucleosome architecture once the genomic response is complete because of the mutation in the critical chromatin remodeling complex. This prolonged remodeled state extends the window of opportunity allowing inappropriate genome regulatory factors access to the genome. Redundant and compensatory chromatin remodeling complexes eventually return the genome to its basal nucleosomal occupancy, but only after the inappropriate loading of regulatory factors has altered the gene expression and fate of the cell. The altered gene expression and fate of the cell may be evident in transformed nucleosome sensitivity patterns. This model reconciles observations across multiple systems and clarifies a role for chromatin remodelers in cancer progression. We suggest that time-resolved measurements of nucleosome occupancy and sensitivity will prove to be valuable biomarkers to characterize cancer progression and will ultimately play a role in the diagnosis and treatment of cancer in the near future.

## References

[1] Kornberg RD, Lorch Y. Twenty-five years of the nucleosome, fundamental particle of the eukaryote chromosome. Cell. 1999 Aug 6; 98(3):285–94. 10458604 10.1016/s0092-8674(00)81958-3

[2] Almer A, Rudolph H, Hinnen A, Hörz W. Removal of positioned nucleosomes from the yeast PHO5 promoter upon PHO5 induction releases additional upstream activating DNA elements. The EMBO Journal. 1986 Oct; 5(10):2689–96. 3536481 10.1002/j.1460-2075.1986.tb04552.x PMC1167170

[3] Almer A, Hörz W. Nuclease hypersensitive regions with adjacent positioned nucleosomes mark the gene boundaries of the PHO5/PHO3 locus in yeast. The EMBO Journal. 1986 Oct; 5(10):2681–7. 3023055 10.1002/j.1460-2075.1986.tb04551.x PMC1167169

[4] Han M, Grunstein M. Nucleosome loss activates yeast downstream promoters in vivo. Cell. 1988 Dec 23; 55(6):1137–45. 2849508 10.1016/0092-8674(88)90258-9

[5] Richard-Foy H, Sistare FD, Riegel AT, Simons SS Jr, Hager GL. Mechanism of dexamethasone 21-mesylate antiglucocorticoid action: II. Receptor-antiglucocorticoid complexes do not interact productively with mouse mammary tumor virus long terminal repeat chromatin. Molecular Endocrinology. 1987 Sep 1; 1(9):659–65. 2856414 10.1210/mend-1-9-659

[6] Richard-Foy H, Hager GL. Sequence-specific positioning of nucleosomes over the steroid-inducible MMTV promoter. The EMBO Journal. 1987 Aug; 6(8):2321–8. 2822386 10.1002/j.1460-2075.1987.tb02507.x PMC553635

[7] Dennis JH, Fan HY, Reynolds SM, Yuan G, Meldrim JC, Richter DJ, Independent and complementary methods for large-scale structural analysis of mammalian chromatin. Genome Research. 2007 Jun 1; 17(6):928–39. 17568008 10.1101/gr.5636607 PMC1891351

[8] Yuan GC, Liu YJ, Dion MF, Slack MD, Wu LF, Altschuler SJ, Genome-scale identification of nucleosome positions in S. cerevisiae. Science. 2005 Jul 22; 309(5734):626–30. 15961632 10.1126/science.1112178

[9] Ozsolak F, Song JS, Liu XS, Fisher DE. High-throughput mapping of the chromatin structure of human promoters. Nature Biotechnology. 2007 Feb; 25(2):244–8. 10.1038/nbt1279 17220878

[10] Sexton BS, Avey D, Druliner BR, Fincher JA, Vera DL, Grau DJ, The spring-loaded genome: nucleosome redistributions are widespread, transient, and DNA-directed. Genome Research. 2014 Feb 1; 24(2):251–9. 24310001 10.1101/gr.160150.113 PMC3912415

[11] Druliner BR, Vera D, Johnson R, Ruan X, Apone LM, Dimalanta ET, Comprehensive nucleosome mapping of the human genome in cancer progression. Oncotarget. 2016 Mar 22; 7(12):13429. 26735342 10.18632/oncotarget.6811 PMC4924652

[12] Druliner B, Fincher J, Sexton B, Vera D, Roche M, Lyle S, Chromatin patterns associated with lung adenocarcinoma progression. Cell cycle. 2013 May 15; 12(10):1536–43. 23598721 10.4161/cc.24664 PMC3680533

[13] Pulivarthy SR, Lion M, Kuzu G, Matthews AG, Borowsky ML, Morris J, Regulated large-scale nucleosome density patterns and precise nucleosome positioning correlate with V (D) J recombination. Proceedings of the National Academy of Sciences. 2016 Oct 18; 113(42):E6427–36. 10.1073/pnas.1605543113 PMC5081657 27698124

[14] Brown AN, Vied C, Dennis JH, Bhide PG. Nucleosome repositioning: a novel mechanism for nicotine-and cocaine-induced epigenetic changes. PloS One. 2015 Sep 28; 10(9):e0139103. 26414157 10.1371/journal.pone.0139103 PMC4586372

[15] Comoglio F, Simonatto M, Polletti S, Liu X, Smale ST, Barozzi I, Dissection of acute stimulus-inducible nucleosome remodeling in mammalian cells. Genes & Development. 2019 Sep 1; 33(17–18):1159–74. 31371436 10.1101/gad.326348.119 PMC6719622

[16] Wei J, Alfajaro MM, DeWeirdt PC, Hanna RE, Lu-Culligan WJ, Cai WL, Genome-wide CRISPR screens reveal host factors critical for SARS-CoV-2 infection. Cell. 2021 Jan 7; 184(1):76–91. 33147444 10.1016/j.cell.2020.10.028 PMC7574718

[17] Lei X, Dong X, Ma R, Wang W, Xiao X, Tian Z, Activation and evasion of type I interferon responses by SARS-CoV-2. Nature Communications. 2020 Jul 30; 11(1):1–2. 10.1038/s41467-020-17665-9 PMC7392898 32733001

[18] Zheng Y, Liu X, Le W, Xie L, Li H, Wen W, A human circulating immune cell landscape in aging and COVID-19. Protein & Cell. 2020 Oct; 11(10):740–70. 32780218 10.1007/s13238-020-00762-2 PMC7417788

[19] Dingwall C, Lomonossoff GP, Laskey RA. High sequence specificity of micrococcal nuclease. Nucleic Acids Research. 1981 Jun 25;9(12):2659–74. 6269057 10.1093/nar/9.12.2659 PMC326883

[20] Vera DL, Madzima TF, Labonne JD, Alam MP, Hoffman GG, Girimurugan SB, Differential nuclease sensitivity profiling of chromatin reveals biochemical footprints coupled to gene expression and functional DNA elements in maize. The Plant Cell. 2014 Oct 1;26(10):3883–93. 25361955 10.1105/tpc.114.130609 PMC4247582

[21] Luger K, Richmond TJ. DNA binding within the nucleosome core. Current Opinion in Structural Biology. 1998 Feb 1;8(1):33–40. 9519294 10.1016/s0959-440x(98)80007-9

[22] Bao Y, Konesky K, Park YJ, Rosu S, Dyer PN, Rangasamy D, Nucleosomes containing the histone variant H2A. Bbd organize only 118 base pairs of DNA. The EMBO Journal. 2004 Aug 18;23(16):3314–24. 15257289 10.1038/sj.emboj.7600316 PMC514500

[23] Mieczkowski J, Cook A, Bowman SK, Mueller B, Alver BH, Kundu S, MNase titration reveals differences between nucleosome occupancy and chromatin accessibility. Nature Communications. 2016 May 6;7(1):1–1. 10.1038/ncomms11485 PMC4859066 27151365

[24] Cole L, Dennis J. MNase Profiling of Promoter Chromatin in Salmonella typhimurium-Stimulated GM12878 Cells Reveals Dynamic and Response-Specific Nucleosome Architecture. G3: Genes, Genomes, Genetics. 2020 Jul 1;10(7):2171–8. 32404364 10.1534/g3.120.401266 PMC7341138

[25] Oruba A, Saccani S, van Essen D. Role of cell-type specific nucleosome positioning in inducible activation of mammalian promoters. Nature Communications. 2020 Feb 26;11(1):1–8. 10.1038/s41467-020-14950-5 PMC7044431 32103026

[26] Winkles JA. Serum-and polypeptide growth factor-inducible gene expression in mouse fibroblasts. Progress in Nucleic Acid Research and Molecular Biology. 1997 Jan 1;58:41–78. 10.1016/s0079-6603(08)60033-1 9308363

[27] Clapier CR, Iwasa J, Cairns BR, Peterson CL. Mechanisms of action and regulation of ATP-dependent chromatin-remodelling complexes. Nature Reviews Molecular Cell Biology. 2017 Jul;18(7):407–22. 28512350 10.1038/nrm.2017.26 PMC8127953

[28] Gonzalez-Perez A, Jene-Sanz A, Lopez-Bigas N. The mutational landscape of chromatin regulatory factors across 4,623 tumor samples. Genome Biology. 2013 Sep;14(9):1–5. 10.1186/gb-2013-14-9-r106 PMC4054018 24063517

[29] Okawa R, Banno K, Iida M, Yanokura M, Takeda T, Iijima M, Aberrant chromatin remodeling in gynecological cancer. Oncology Letters. 2017 Nov 1;14(5):5107–13. 29113150 10.3892/ol.2017.6891 PMC5656032

[30] Kumar R, Li DQ, Müller S, Knapp S. Epigenomic regulation of oncogenesis by chromatin remodeling. Oncogene. 2016 Aug;35(34):4423–36. 26804164 10.1038/onc.2015.513

[31] Chen J, Herlong FH, Stroehlein JR, Mishra L. Mutations of chromatin structure regulating genes in human malignancies. Current Protein and Peptide Science. 2016 Aug 1;17(5):411–37. 26796307 10.2174/1389203717666160122120008 PMC5403969

[32] Skulte KA, Phan L, Clark SJ, Taberlay PC. Chromatin remodeler mutations in human cancers: epigenetic implications. Epigenomics. 2014 Aug;6(4):397–414. 25333849 10.2217/epi.14.37

